# miR-146a Deficiency Accelerates Hepatic Inflammation Without Influencing Diet-induced Obesity in Mice

**DOI:** 10.1038/s41598-019-49090-4

**Published:** 2019-09-02

**Authors:** Aida Javidan, Weihua Jiang, Michihiro Okuyama, Devi Thiagarajan, Lihua Yang, Jessica J. Moorleghen, Latha Muniappan, Venkateswaran Subramanian

**Affiliations:** 1 Saha Cardiovascular Research Center, University of Kentucky, Lexington, KY USA; 2 Department of Physiology, University of Kentucky, Lexington, KY USA

**Keywords:** Metabolic syndrome, Obesity

## Abstract

miR-146a, an anti-inflammatory microRNA, is shown to be a negative regulator of adipocyte inflammation. However, the functional contribution of miR-146a in the development of obesity is not defined. In order to determine whether miR-146a influences diet-induced obesity, mice that were either wild type (WT) or miR-146a deficient (KO) were fed with high (60% kcal) fat diet (HFD) for 16 weeks. Deficiency of miR-146a did not influence obesity measured as HFD-induced body weight and fat mass gain, or metabolism of glucose and insulin tolerance. In addition, adipocyte apoptosis, adipose tissue collagen and macrophage accumulation as detected by TUNEL, Picro Sirius and F4/80 immunostaining, respectively, were comparable between the two groups of mice. Although, miR-146a deficiency had no influence on HFD-induced hepatic lipid accumulation, interestingly, it significantly increased obesity-induced inflammatory responses in liver tissue. The present study demonstrates that miR-146a deficiency had no influence on the development of HFD-induced obesity and adipose tissue remodeling, whereas it significantly increased hepatic inflammation in obese mice. This result suggests that miR-146a regulates hepatic inflammation during development of obesity.

## Introduction

Obesity is characterized by persistent and chronic low grade adipose tissue inflammation, which in turn promotes insulin resistance and metabolic syndrome^1,2^.

In addition, obesity is also strongly associated with the development of non-alcoholic fatty liver disease (i.e.) liver steatosis and inflammation^3,4^. However, mechanisms underlying the process of adipose tissue inflammation and hepatic steatosis during obesity development remain unknown. Recently, microRNAs (miRNAs), a class of small non-coding RNAs have emerged as an important regulator of adipose tissue function in obesity^5^. miRNAs are also shown to suppress insulin resistance in animal models by improving adipose tissue endothelial function^6,7^. In addition, miRNAs also regulates hepatic lipid metabolism and alleviates non-alcoholic fatty liver disease in diet-induced obese mice^8,9^. miRNAs play a crucial role in regulating mRNA stability and translational repression, leading to the modulation of ~30% of the human genome^10^. miR-146a, a member of miR-146 family is shown to play a critical role in immune responses under various disease conditions in humans and mice^11–13^. miR-146a is shown to regulate immune response by suppressing the TLR4 mediated NF-kB signaling pathway^14^. Recently, it has been reported that miR-146a is upregulated in adipose tissue from both obese humans and mice^15,16^. In addition, in cultured human adipocytes, mimetic-mediated overexpression of miR-146a suppressed adipocyte inflammation by targeting IRAK1 and TRAF6^15^. However, the functional contribution of miR-146a in adipose tissue inflammation and remodeling, glucose and insulin tolerance, and hepatic steatosis during the development of obesity remains to be elucidated.

Using miR-146a deficient mice, in the present study, we examined the contribution of miR-146a to the development of diet-induced obesity (body weight gain), adipose tissue inflammation, remodeling (e.g. fibrosis, apoptosis, macrophage accumulation), glucose and insulin tolerance, hepatic steatosis and inflammation in mice. Our study demonstrates that miR-146a deficiency in mice had no influence on the development of high fat diet (HFD)-induced (i) obesity (body weight gain), (ii) adipose tissue inflammation, (iii) adipose tissue remodeling (iv) glucose and insulin tolerance. Interestingly, miR-146a deficiency strongly increased inflammation in the liver tissue without influencing lipid deposition in the liver.

## Results

### High fat diet-induced obesity in mice increased mir-146a expression in adipose tissue

**Figure 1:**
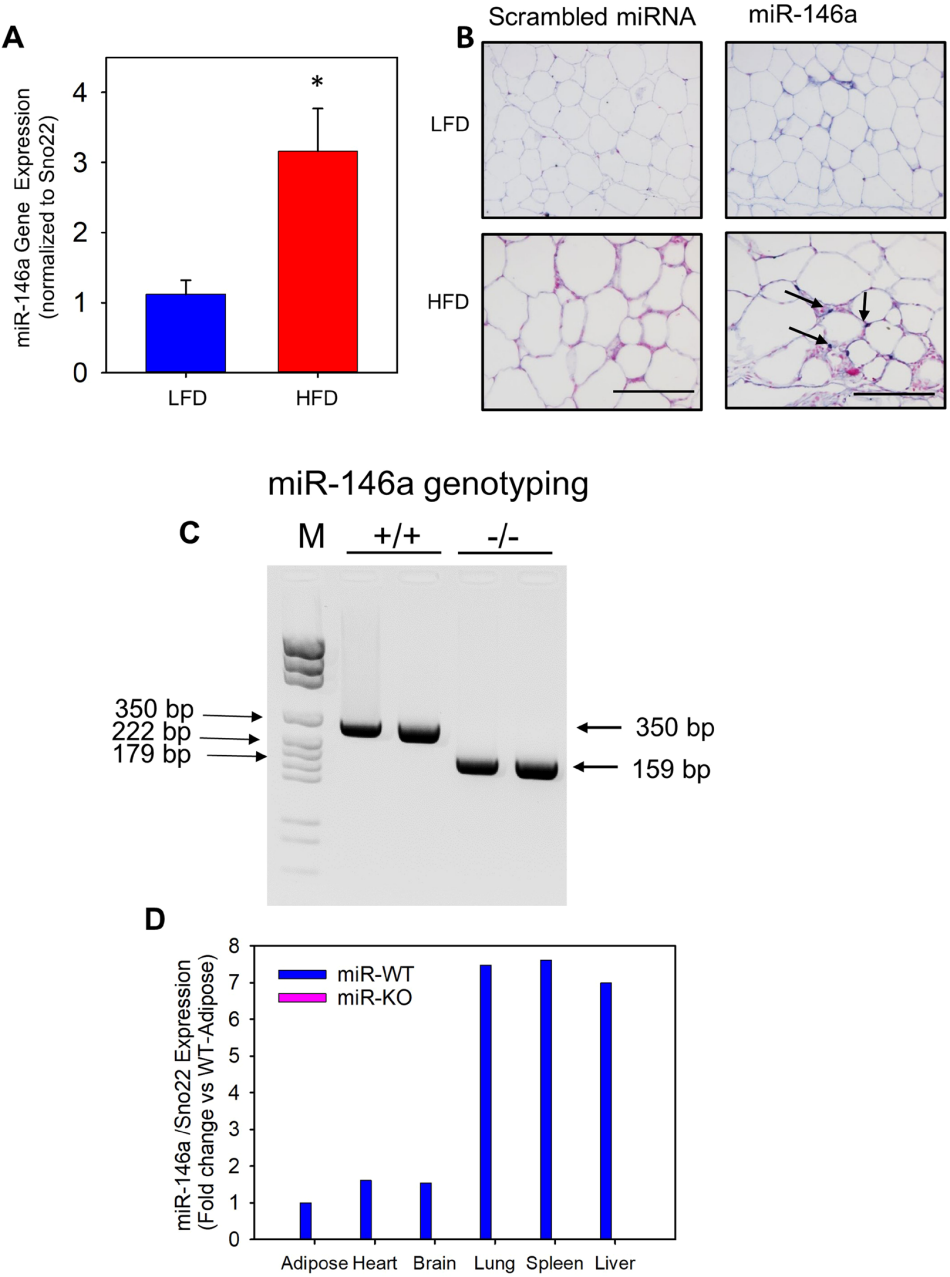
Abundance of miR-146a was increased in obese adipose tissue. (**A**) miR-146a expression was detected by qPCR in epididymal white adipose tissue (EpiWAT) of wild type mice fed either a LFD or HFD for 16 weeks (n = 9–10). (**B**) *In-situ* hybridization of miR-146a in cross-sections from the EpiWAT of LFD and HFD fed wild type mice (bluish-purple color). Arrows indicate a positive miR-146a signal. (**C**) Genomic DNA from tail was isolated and screened by PCR for the miR-146a allele. PCR on tail DNA yielded amplicons of 350 and 159 bp for miR-146a WT and KO alleles, respectively. (M = Molecular weight ladder). Representative PCR gel image is cropped from the full-length gel image. The full-length gel image is included in the Supplementary information. (**D**) miR-146a expression in various tissues of miR-146a WT and KO mice was measured by qPCR. Values are represented as mean ± SEM. * represents significance of *P* < 0.05 (Mann-Whitney Rank Sum test).

In order to understand whether miR-146a expression is influenced by obesity in adipose tissue, adipose tissue mRNA from male mice (C57BL/6) fed either a 10% Kcal low fat diet (LFD) or a 60% Kcal high fat diet (HFD) for 16 weeks. miR qPCR analyses using Taqman primers against miR-146a and snoRNA202 (internal control) showed that miR-146a abundance was significantly increased in epididymal adipose tissue from HFD-induced obese mice compared to LFD fed mice (Fig. 1A). In addition, *in situ* hybridization of adipose tissue using digoxigenein labelled LNA detection probe complimentary to miR-146a or scrambled miRNA (negative control) also confirmed increased miR-146a expression in HFD mice when compared to lean mouse adipose tissue (Fig. 1B).

### High fat diet-induced body weight gain and fat mass are not influenced by mir-146a deficiency in mice

To look into the role of miR-146a in diet-induced obesity, 8 weeks old female C57BL/6J mice that are either miR-146a proficient (WT) or deficient (KO) were used. First, we confirmed absence of the miR-146a gene by PCR using tail genomic DNA (Fig. 1C). miR-qPCR analyses of diverse tissues such as adipose, heart, brain, lung, spleen and liver showed a complete non-existence of miR-146a in the miR-146a KO mice compared to miR-146a WT littermate controls (Fig. 1D). This data confirms absence of miR-146a in diverse tissues.

**Figure 2:**
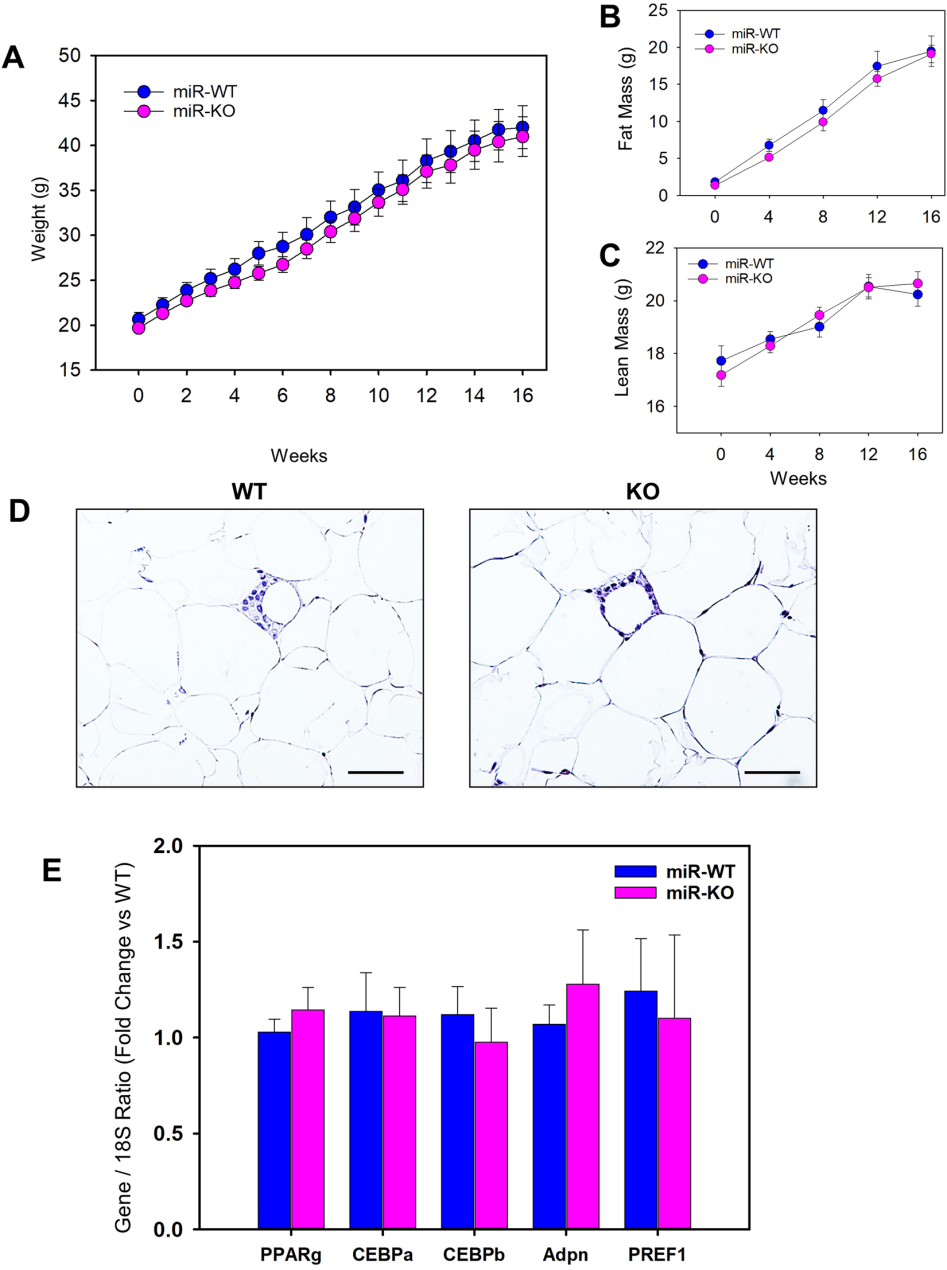
miR-146a deficiency had no effect on diet-induced body weight gain and fat mass in mice. (**A**) Weekly body weight of HFD fed female miR-146a WT and KO mice (n = 11). Monthly body fat mass (**B**) and lean mass (**C**) measurements of HFD fed female miR-146a WT and KO mice (n = 11). (**D**) Representative hematoxylin and eosin stained EpiWAT cross-sections from HFD fed miR-146a WT and KO mice. mRNA abundance of PPARγ, CEBPα, CEBPβ, adiponectin, and PREF-1 genes in EpiWAT from HFD fed female miR-146a WT and KO mice were analyzed by qPCR (n = 7–11). Values are represented as mean ± SEM. Statistical significance were analyzed by Student’s *t* test or Mann-Whitney Rank Sum test.

To explore the role of miR-146a on body weight gain under obese condition, female miR-WT or KO mice were fed with 60% Kcal diet for 16 weeks. miR-146a deficiency had no influence on diet-induced body weight gain, as both WT and KO mice showed a similar and equivalent increase in body weight compared to baseline (week 0) measurements (Fig. 2A). Similarly, there is no significant difference between the 2 genotypes on the increase of HFD-induced body fat composition such as fat (Fig. 2B) and lean mass (Fig. 2C). The mass of adipose tissues such as subcutaneous, epididymal, retroperitoneal, and brown adipose, and liver were comparable between the two groups of mice fed with HFD (Table 1). Similarly, in a small cohort of male mice, we tested the outcome of miR-146a deficiency on body weight and fat mass composition. Similar to females, male mice also showed no significant difference between two genotypes in HFD increased body weight and fat mass (Figure SI in the online-only Data Supplement). Staining of epididymal adipose tissue cross-sections with hematoxylin and eosin showed that adipocyte size are equivalently increased upon HFD feeding from mice of each WT and KO genotype (Fig. 2D). Furthermore, mRNA abundance of genes involved in adipogenesis (e.g. PPARγ, CEBP, adiponectin, PREF-1) were equivalent between the two genotypes of HFD fed mice (Fig. 2E). Therefore, miR-146a deficiency had no influence on diet-induced obesity development in HFD fed mice.

**Table 1:** Effects of miR-146a deficiency on liver and fat pad weight in HFD fed female mice.

Groups	miR-146a WT	miR-146a KO
N	11	11
Liver Weight (g)	1.6 ± 0.19	1.5 ± 0.13
Epididymal Adipose (g)	2.1 ± 0.27	2.2 ± 0.26
Retroperitoneal Adipose (g)	0.5 ± 0.10	0.3 ± 0.04
Sub-cutaneous Adipose (g)	1.6 ± 0.28	1.6 ± 0.23
Brown Adipose (g)	0.2 ± 0.03	0.2 ± 0.02

Values are represented as means ± SEMs. *Denotes *P* < 0.05 when comparing WT vs KO, by student’s t test.

### miR-146a does not contribute to glucose and insulin tolerance in obese mice

**Figure 3:**
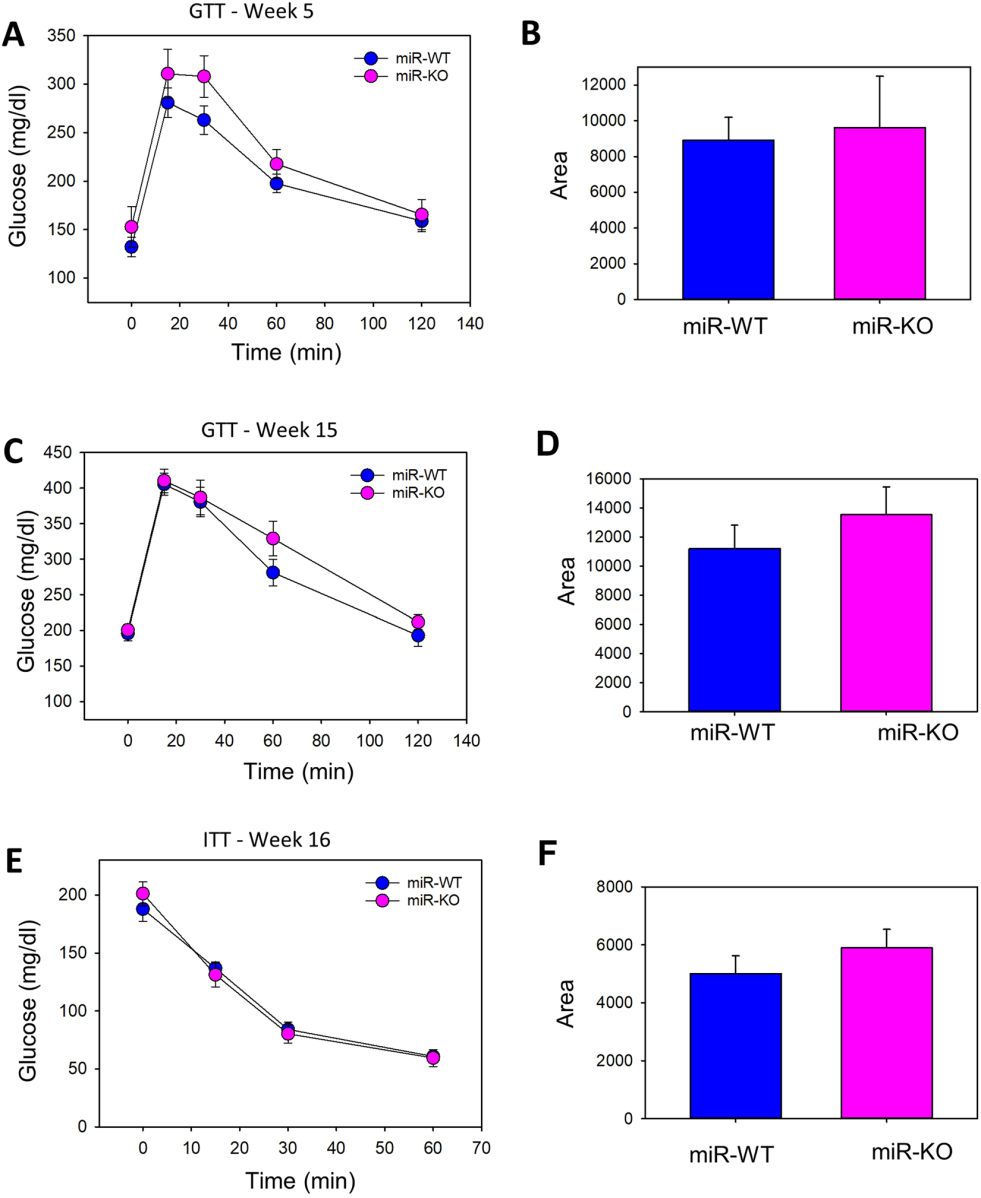
miR-146a does not contribute to glucose and insulin tolerance in obese mice. Glucose tolerance test (GTT) and the area under curve (AUC) of GTT at week 5 (**A**,**B**) and week 15 (**C**,**D**) during HFD consumption in female miR-146a WT and KO mice. Insulin tolerance test (ITT) and the AUC of ITT at week 16 (**E**,**F**) post HFD in female miR-146a WT and KO mice (n = 11). Values are represented as mean ± SEM. Statistical significance were analyzed by Student’s *t* test or Mann-Whitney Rank Sum test.

To identify the possible role of miR-146a deficiency on glucose tolerance in mice under obese condition, glucose tolerance tests (GTT) were performed during at 5 weeks (early stage) and 15 weeks (late stage) and late (15 weeks of diet) stage of obesity. Upon HFD feeding, miR-146a deficient mice showed no improvement in glucose tolerance both at early (Fig. 3A,B) and late stages of obesity (Fig. 3C,D). Similar to GTT, miR-146a deficiency had no influence on HFD feeding impaired insulin tolerance tests (ITT), in both groups of mice (Fig. 3E,F). Similar to female mice, miR-146a deficiency had no influence on GTT and ITT in HFD fed male mice. (Fig. SII in the online-only Data Supplement). Plasma insulin concentrations were equal in both miR-146a-WT and KO groups of mice (Fig. SIII in the online-only Data Supplement).

### miR-146a deficiency does not affect high fat diet-induced adipocyte cell death

**Figure 4:**
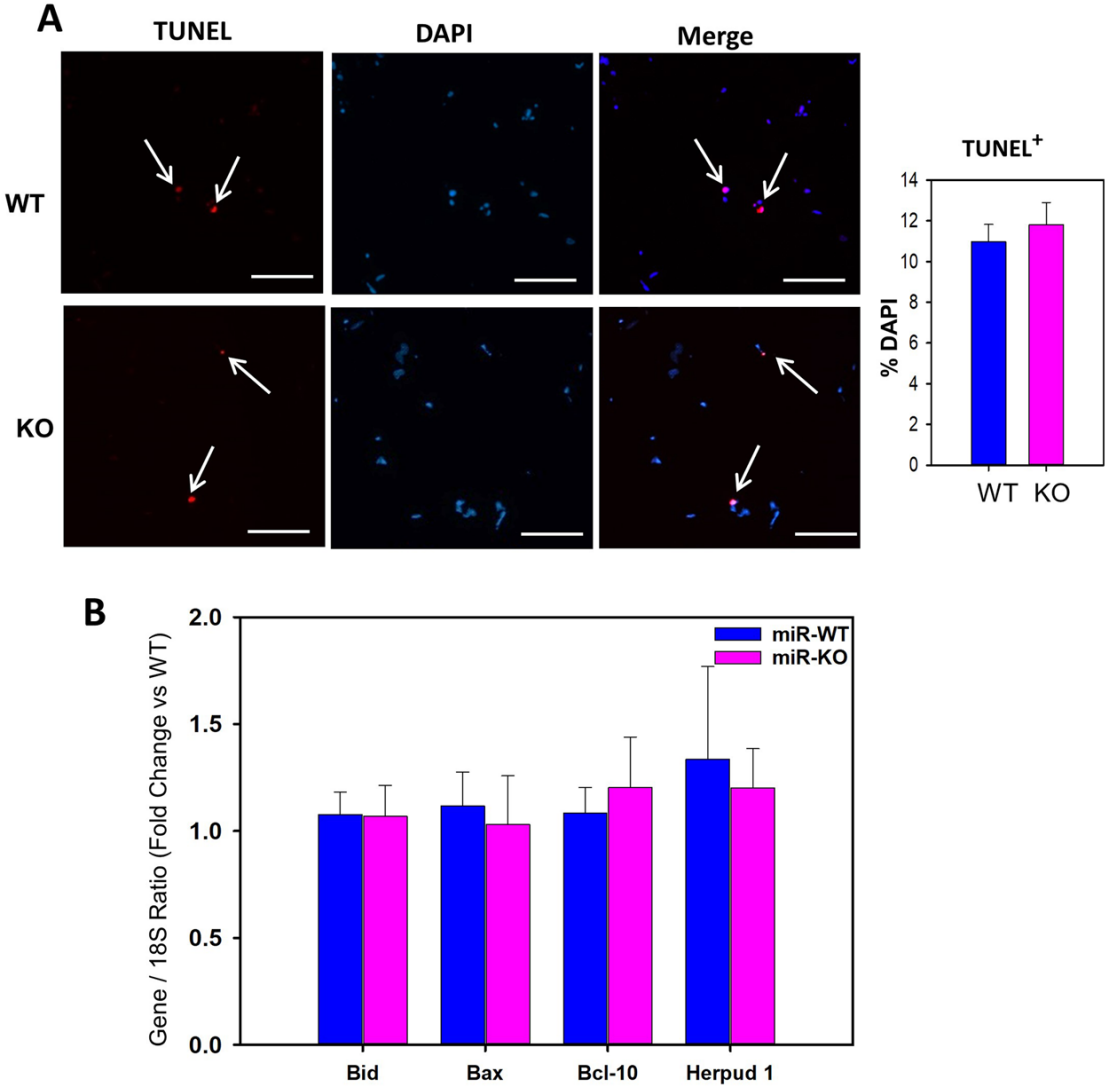
miR-146a does not affect obesity-induced adipocyte cell death. (**A**) Representative TUNEL staining of EpiWAT cross-sections and quantification graph from 16 weeks HFD fed miR-146a WT and KO mice. Nuclei were stained with DAPI (blue) and TUNEL-positive cells (red) are indicated by arrows. Under fluorescent microscopy, TUNEL-positive cells were counted from 10 fields at the power of 100x magnification (n = 5). (**B**) mRNA abundance of Bid, Bax, Bcl10 and Herpud1 genes in EpiWAT from HFD fed miR-146a WT and KO mice were analyzed by qPCR (n = 7–11). Values are represented as mean ± SEM. Statistical significance were analyzed by Student’s *t* test or Mann-Whitney Rank Sum test.

Increased apoptosis mediated adipocyte cell death during obesity, triggers phagocytosis, by infiltrated macrophages, to remove the cell debris in adipose tissue^17,18^. To test whether miR-146a deficiency had any role on apoptosis-mediated adipocyte cell death under HFD (16 weeks) condition, TUNEL staining was performed on cross-sections of EpiWAT from WT and miR-146a deficient groups of mice. TUNEL-positive (apoptotic) cells were clearly observed in adipose tissue after HFD feeding, but equivalent in both WT and miR-146a deficient groups of mice (Fig. 4A). Correspondingly, in adipose tissue, mRNA abundance of genes involved in apoptosis (e.g. Bid, Bax, BCL10) were comparable between the two groups of mice (Fig. 4B).

### miR-146a deficiency in mice does not influence obesity accelerated macrophage accumulation and inflammation in adipose tissue

**Figure 5:**
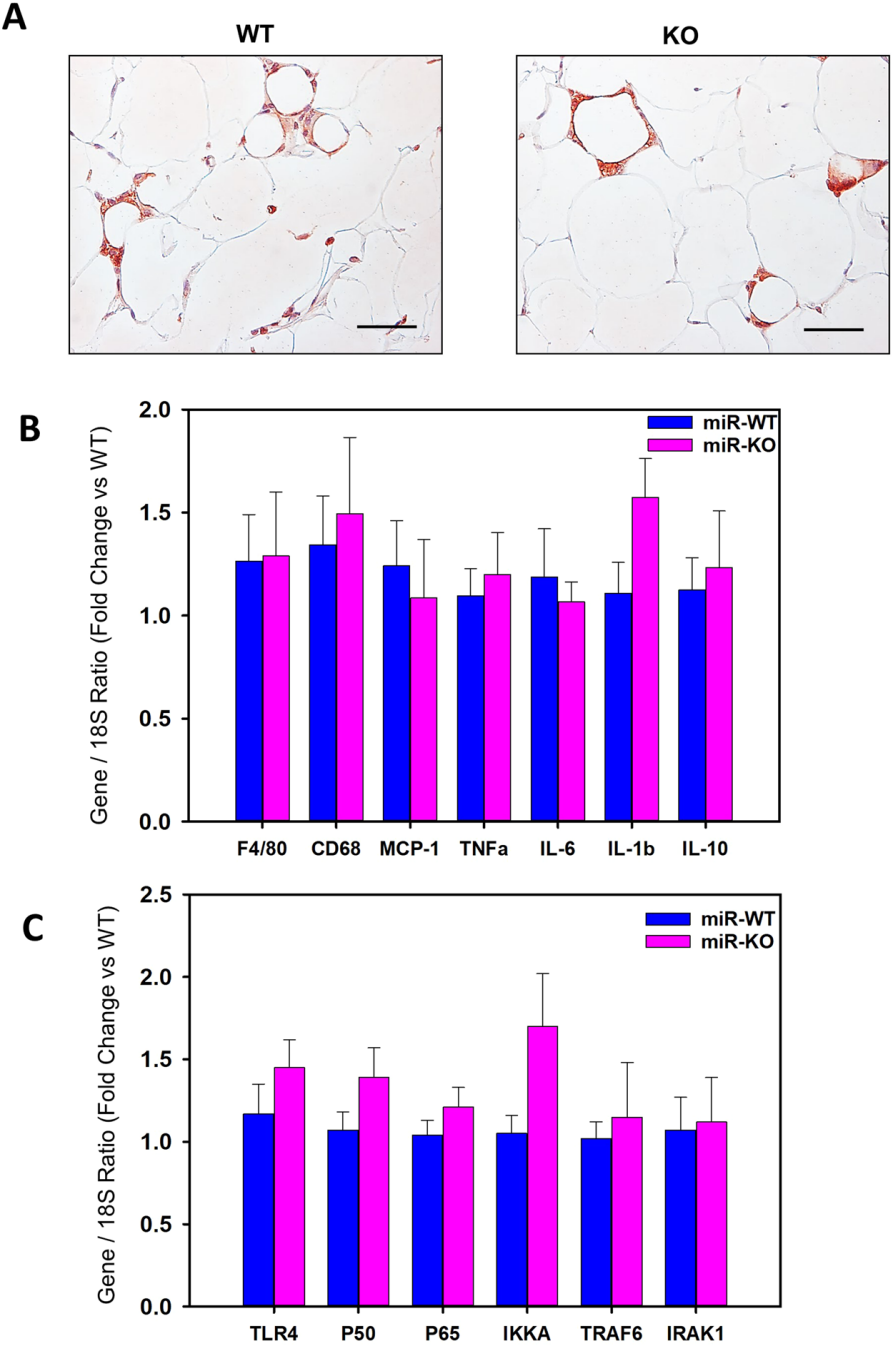
miR-146a deficiency does not influence adipose tissue macrophage accumulation and inflammation in obese mice. (**A**) Representative immunohistochemical staining of F4/80 (red color) in EpiWAT cross-sections from 16 weeks HFD fed miR-146a WT and KO mice. (**B**) mRNA abundance of F4/80, CD68, MCP-1, TNFα, IL-6, IL-1β and IL-10 in EpiWAT from HFD fed miR-146a WT and KO mice were analyzed by qPCR (n = 7–11). (**C**) mRNA abundance of TLR4, NF-kB (P50), RelA (P65), IKKα, TRAF6 and IRAK1 in EpiWAT from HFD fed miR-146a WT and KO mice were analyzed by qPCR (n = 5–11). Values are represented as mean ± SEM. Statistical significance were analyzed by Student’s *t* test or Mann-Whitney Rank Sum test.

Under obese condition, adipocyte apoptosis is one of the key event that promotes macrophage infiltration into adipose tissue^18^. Here, we tested the involvement of miR-146a on HFD-induced obesity accelerated macrophage accumulation in adipose tissue. Immunostaining using antibodies against F4/80 revealed accumulation of F4/80+ macrophages in obese adipose tissue, but comparable in both WT and miR-146a deficient mice (Fig. 5A). Correspondingly, mRNA abundance of macrophage marker genes such as F4/80 and CD68, and monocyte chemoattractant protein-1 (MCP-1) were comparable between the two groups. Furthermore, mRNA abundance of pro-inflammatory marker genes (TNFα, IL-1β and IL-6) and anti-inflammatory IL-10 gene, were not influenced by miR-146a deficiency (Fig. 5B). Since miR-146a known to regulate inflammatory TLR4/NF-kB signaling, next we explored the possible effect of miR-146a deficiency on mRNA expression of these inflammatory cascade genes. qPCR analyses detected no differences in mRNA abundance of TLR4, NF-kB P50, P65 and IKKα between WT and miR-146a deficient mice (Fig. 5C). In addition, miR-146a deficiency had no effect on mRNA abundance of its known putative targets such as TRAF6 and IRAK1 in adipose tissue (Fig. 5C).

### miR-146a does not contribute adipose tissue fibrosis in obese mice

**Figure 6:**
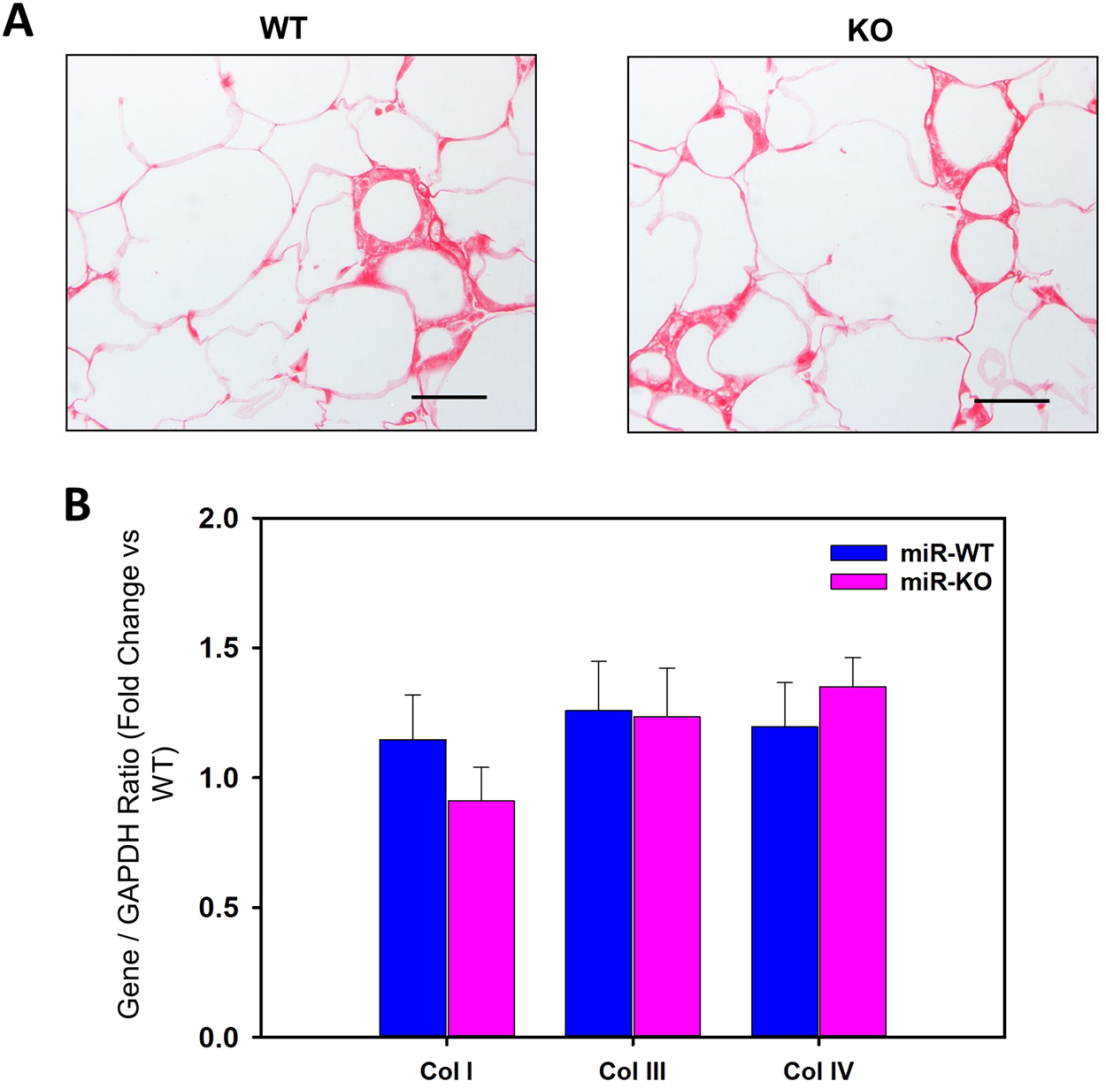
miR-146a does not contribute to HFD-induced adipose tissue fibrosis. (**A**) Representative Sirius red staining of EpiWAT cross-sections from HFD fed miR-146a WT and KO mice. (**B**) mRNA abundance of Col I, Col III and Col IV genes in EpiWAT from HFD fed miR-146a WT and KO mice were analyzed by qPCR (n = 10–11). Values are represented as mean ± SEM. Statistical significance were analyzed by Student’s *t* test or Mann-Whitney Rank Sum test.

Recently, in obese individuals, increased extracellular matrix material accumulation and fibrosis in white adipose tissue have been reported^19^. Increased adipose fibrosis during obesity is suggested to enhance dysfunction of adipose tissue^20^. To reveal the contribution of miR-146a on adipose fibrosis, picro-sirius red staining was performed on cross-sections of EpiWAT from 16 weeks HFD fed WT and miR-146a deficient mice. HFD feeding increased adipose tissue fibrosis equivalently in both WT and miR-146a deficient mice (Fig. 6A). Correspondingly, qPCR analyses showed mRNA abundance of collagen genes (I, III and IV) induced by HFD feeding were not affected by miR-146a deficiency compared to WT group (Fig. 6B).

### miR-146a deficiency does not affect hfd-induced liver steatosis, but increased inflammation

**Figure 7:**
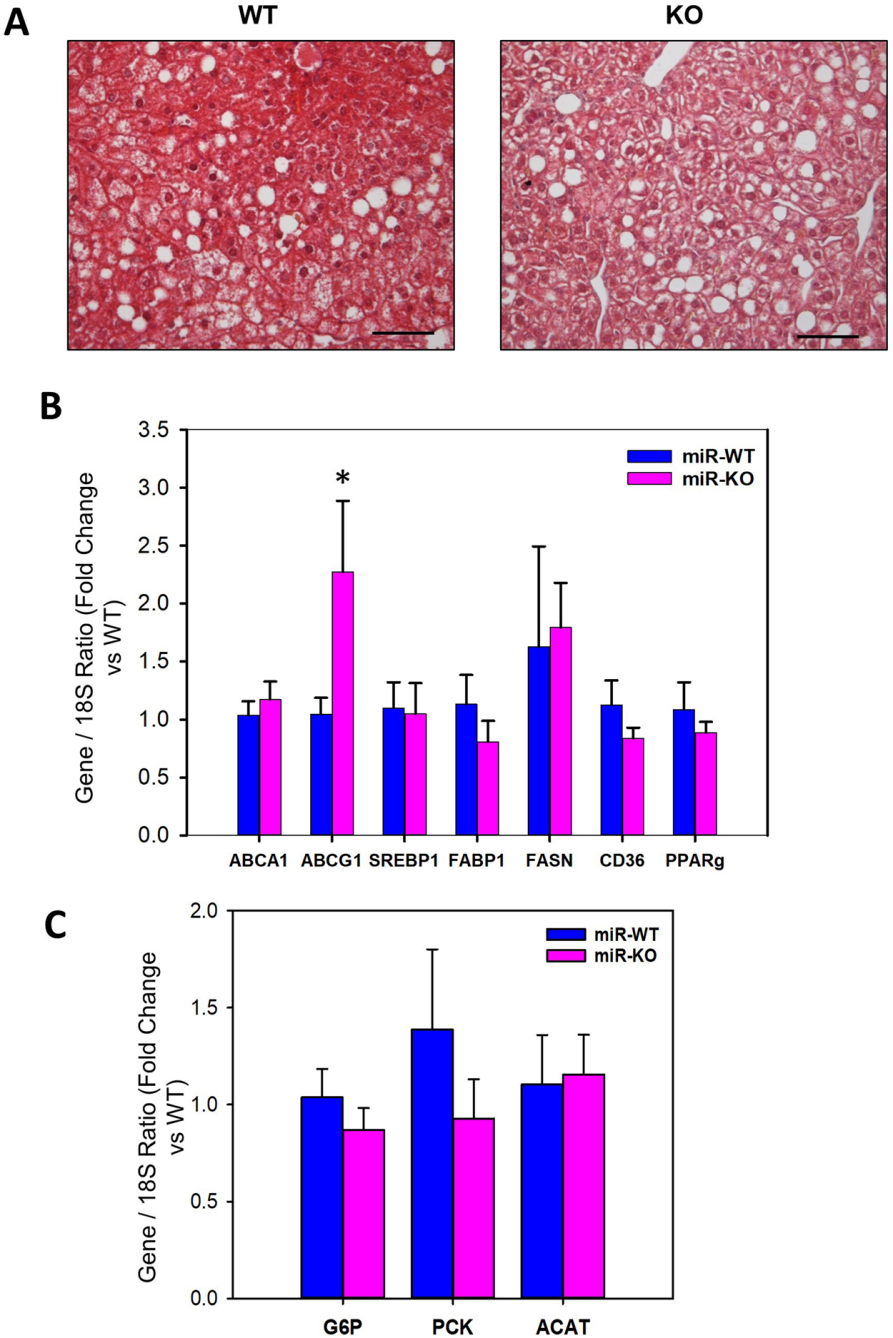
miR-146a deficiency had no effect on HFD-induced liver steatosis. (**A**) Representative hematoxylin and eosin staining of liver cross-sections from HFD fed miR-146a WT and KO mice. (**B**) mRNA abundance of ABCA1, ABCG1, SREBP1, FABP1, FAS, CD36 and PPARγ genes in liver from 16 weeks HFD fed miR-146a WT and KO mice. (**C**) mRNA abundance of G6P, PCK-1 and ACAT genes in liver from HFD fed miR-146a WT and KO mice. mRNA abundance of various genes were analyzed by qPCR (n = 10–11). Values are represented as mean ± SEM. *Denotes *P* < 0.05 when comparing WT vs KO (Student’s *t* test or Mann-Whitney Rank Sum test).

Histological studies on liver cross-section from WT and miR-146a deficient obese mice were performed to assess whether miR-146a exert any role on other metabolic organ such as liver. Hematoxylin and eosin staining showed that HFD diet feeding accelerated steatosis – lipid deposition, similarly in both groups (Fig. 7A). In addition, analyses of lipids extracted from liver showed no differences in hepatic cholesterol and triglycerides. (Fig. SIV in the online-only Data Supplement). Furthermore, mRNA abundance of selected genes in liver tissue were comparable between WT and KO groups (Fig. 7B). miR-146a deficiency had no significant effect on fatty acid synthase (FAS), fatty acid binding protein (FABP), CD36, PPARγ, and lipid transporter, ABCA1 gene expression, whereas it showed a significant increase in mRNA abundance of lipid transporter, ABCG1, in liver. In addition, glucose metabolic genes such as glucose-6-phosphatase (G6P) and phosphoenolpyruvate carboxykinase 1 (PCK-1) and acyl-CoA: cholesterol acyltransferase (ACAT) were not influenced in liver by miR-146a deficiency (Fig. 7C).

**Figure 8:**
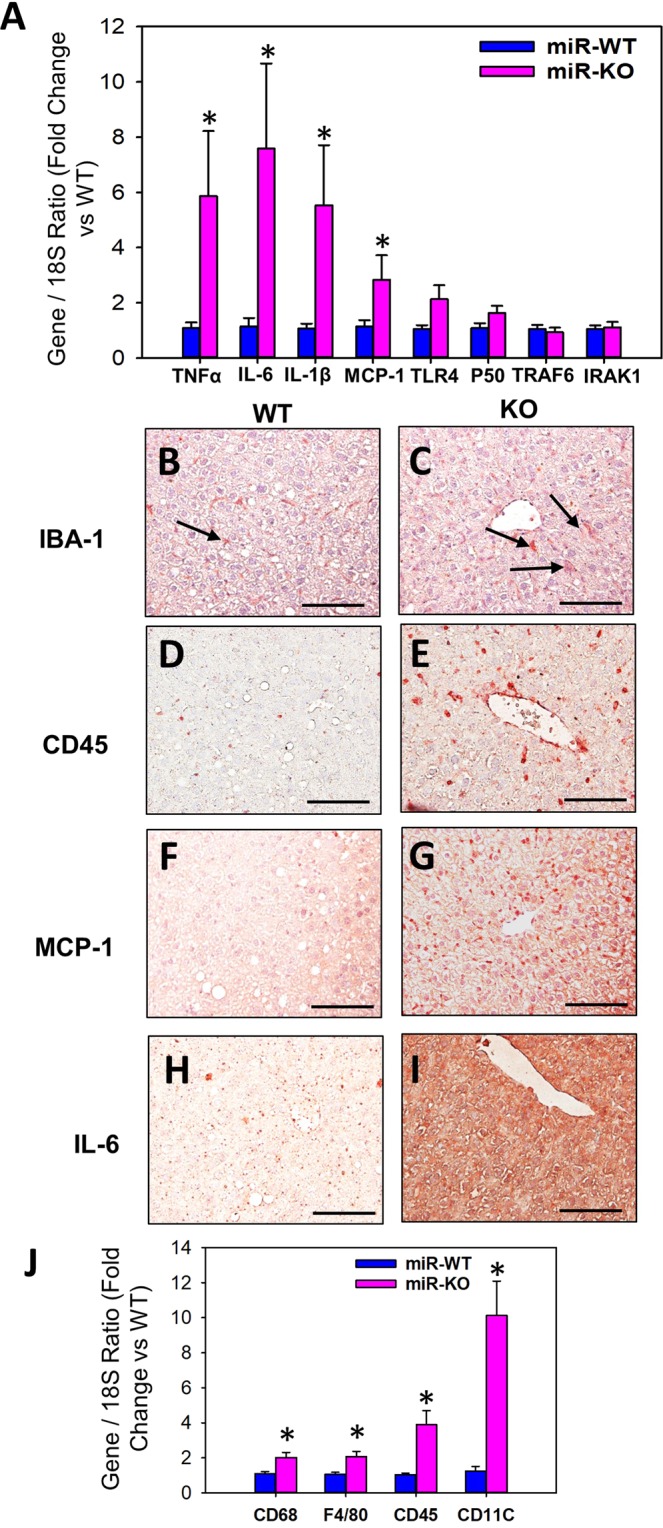
miR-146a deficiency increased HFD-induced hepatic inflammation. (**A**) mRNA abundance of TNF-α, IL-6, IL-1β, MCP-1, TLR4, NF-kB (P50), TRAF6 and IRAK1 genes in liver from HFD fed miR-146a WT and KO mice. Representative serial cross-sections from HFD-fed miR-146a WT and KO mice liver tissue sections immunostained with anti-IBA-1 (**B**,**C**), CD-45 (**D**,**E**), MCP-1 (**F**,**G**) and IL-6 (**H**,**I**). (**J**) mRNA abundance of CD68, F4/80, CD45 and CD11C genes in liver from HFD fed miR-146a WT and KO mice. mRNA abundance of various genes were analyzed by qPCR (n = 10–11). Values are represented as mean ± SEM. *Denotes *P* < 0.05 when comparing WT vs KO (Student’s *t* test or Mann-Whitney Rank Sum test).

With respect to HFD-induced inflammation in liver, miR-146a deficiency significantly increased mRNA abundance of TNFα, IL-6, IL-1β and MCP-1, whereas it had no effect on TLR4, NF-kB P50, and its known putative targets such as TRAF6 and IRAK1 (Fig. 8A). In addition, immunohistochemical staining on liver sections using IBA-1 antibodies revealed HFD feeding increased kupffer cell activation in miR-146a deficient mice compared to WT group (Fig. 8B,C). In addition, miR-146a deficiency showed increased accumulation of CD45 positive leukocytes in the liver compared to WT group (Fig. 8D,E). Furthermore, immunohistochemical staining using MCP-1 and IL-6 antibodies showed strong positive staining in miR-146a deficient liver sections compared to WT liver tissue sections (Fig. 8F–I). With respect to MCP-1, the positive staining is mainly on the activated kupffer cells, and on infiltrated immune cells (Fig. 8F,G), whereas IL-6 positive staining is predominantly by the resident liver cells (Fig. 8H,I). Correspondingly, qPCR analyses also further confirmed that a strong induction of mRNA abundance of leukocyte marker genes such as CD11C, CD45, CD68 and F4/80 in the miR-146a deficient liver compared to WT group (Fig. 8J).

## Discussion

Obesity development is strongly associated with extensive remodeling of adipose tissue including hypertrophy and apoptosis of adipocytes, infiltration and accumulation of macrophages, and adipose tissue fibrosis. By utilizing miR-146a deficient mice, we studied the functional contribution of miR-146a to obesity-accelerated adipose tissue remodeling. Our study revealed no critical role for miR-146a in diet-induced obesity development, as evident by its deficiency which had no effects on adipose tissue remodeling including adipocyte apoptosis and fibrosis, in addition to macrophage accumulation and adipose tissue inflammation. Although miR-146a deficiency did not impact liver steatosis, it strongly increased hepatic inflammation in obese mice.

In the present study, 16 weeks of HFD-induced obesity extremely increased epididymal WAT adipose tissue miR-146a gene expression in mice. Consistent with our current observation, subcutaneous WAT from obese humans also showed an increased miR-146a expression compared to lean subjects^15^. Furthermore, subcutaneous WAT from 8 weeks HFD fed mice also showed an elevated miR-146a expression^15^. Using a mouse model of diet-induced obesity, our present study revealed that miR-146a deficiency had no effect on obesity development. Consistent with this current observation, but in a different model of HFD (42% saturated fat and 0.2% cholesterol) consumption for 16 weeks in LDL receptor deficient mice, deficiency of miR-146a had no influence on body weight gain^21^. However, in cultured porcine subcutaneous adipocytes, inhibition of miR-146a modestly upregulated adipogenesis by increasing triglyceride mediated lipid droplets *in vitro*^22^. Furthermore, glucose and insulin tolerance test studies indicated that obesity associated insulin resistance were exacerbated upon HFD feeding. However, miR-146a deficiency did not affect HFD-induced insulin resistance. In contrast, in cultured 3T3L1 differentiated adipocytes, mimetic mediated overexpression of miR-146a improved glucose uptake^23^. Similarly, in a small population of Asian Indian type 2 diabetics, decreased miR-146a in peripheral blood monocytes are associated with insulin resistance and poor glycemic control^24^. In obese mice, reduction of PCK-1 in liver is shown to accelerate insulin sensitivity^25^. Up on HFD feeding, miR-146a deficiency showed no significant difference on glucose metabolic genes such as PCK-1 and G6P in the liver tissue. Since miR-146a had no influence on HFD-induced obesity, a comparable level of insulin resistance was expected as miR-146a has been described as an important anti-inflammatory miRNA^14,15,26^.

During obesity development, apoptosis-mediated loss of adipocytes has been suggested to be an underlying mechanism that leads to infiltration and accumulation of macrophages in adipose tissue^17,18^. In support, inactivation of pro-apoptotic gene, Bid, in mice, prevented apoptosis-mediated loss of adipocytes and macrophage infiltration into adipose tissue^27^. Mimetic mediated agonism of miR-146a is shown to promote acute lymphoblastic leukemia Jurkat cells death by upregulating STAT1, an apoptosis promoting factor, and suppressing anti-apoptotic Bcl-10^28^. In addition, in murine osteoblastic MC3T3-E1 cells, miR-146a overexpression shown to suppress anti-apoptotic Bcl-2 and promote apoptosis^29^. qPCR analyses of pro-apoptotic genes in Epi-WAT showed comparable expression between miR-146a WT and KO groups of mice, in addition to obesity-induced apoptotic cell death measured by TUNEL assay. Consistent with adipocyte apoptosis, miR-146a deficiency in obese mice showed no influence on macrophage accumulation, and inflammation in adipose tissue. In contrast, in cultured human adipocytes, mimetics mediated overexpression of miR-146a suppressed adipocyte inflammation by targeting TRAF6 and IRAK1^15^. Our qPCR studies ruled out that there is no alteration in TRAF6 and IRAK1 gene expression in EPiWAT of 16 weeks HFD-fed miR-146a KO mice compared to WT mice.

miR-146a, as an anti-inflammatory regulator, has been shown to mediate its function in various immune cells by repressing NF-kB and TLR4 signaling^30^. However, surprisingly, we did not observe any alterations in expression of NF-kB associated P50, P65, TLR4 and other inflammatory genes such as TNFα, IL-6, IL-1β and IL-10 in miR-146a deficient WAT after 16 weeks of HFD compared to WT. One possible explanation is that compensation by another inflammatory micro RNA miR-155, after chronic HFD feeding. In support, a recent study showed that in 12 months old aged miR-146a deficient mice, upon LPS stimulation, a supra-activation of miR-155 expression along with increased IL-6 secretion in bone-marrow derived macrophages^31^. Furthermore, double deficiency of miR-146a and miR-155 in mice rescued miR-146a deficiency enhanced inflammatory responses upon LPS stimulation or *Listeria* infection^31^. In addition, miR-155 deficiency in mice also suppressed WAT inflammation in obese mice in addition to improved insulin resistance^32^. Further studies are warranted to understand whether there is an association between miR-146a and miR-155 in regulating NF-kB and TLR4 signaling mediated inflammation, macrophage accumulation and insulin resistance in adipose tissue in long term obesity condition.

Accumulation of extracellular matrix components such as collagen in adipose tissue has been implicated in systemic insulin resistance and hepatic steatosis in obese individuals and mice^19,33,34^. Literature search with respect to miR-146a and fibrosis revealed that transgenic overexpression of miR-146a in endothelial cells suppressed diabetes induced cardiac fibrosis in mice^35^ and in cultured rat hepatic stellate cells^36^. In the present study, miR-146a deficiency had no influence on adipose tissue fibrosis, and on various collagen genes, which are well known to play a critical role in the production of matrix components in adipose tissue. This finding is well coincides with our observation on adipose tissue macrophage accumulation, in which we did not observe any difference in miR-146a deficient mice compared to WT mice. Accumulated adipose tissue macrophages are recently shown to play a critical role in the regulation of adipose tissue fibrosis^37^.

Recent studies highlighted that chronic inflammation precedes hepatic steatosis in diet-induced obese mice^38^. Since miR-146a is an anti-inflammatory response miRNA, we examined the effect of miR-146a deficiency on hepatic lipid accumulation and inflammation. In spite of increased gene expression of lipid transporter, ABCG1, miR-146a showed no difference on hepatic triglycerides concentrations after 16 weeks of HFD. Similarly, although in a different set of high fat/high cholesterol (21% fat and 1.25% cholesterol) diet condition for 9 weeks, ABCG1 overexpressing transgenic mice showed no effect on triglyceride accumulation in the liver^39^. Surprisingly, in contrast to adipose tissue, miR-146a deficiency showed an accelerated liver inflammation as observed by increased mRNA abundance of TNFα, IL-6, IL-1β and MCP-1 without influencing TLR4, NF-kB P50 and its putative targets, TRAF6 and IRAK1. In addition, immunohistochemical staining analyses showed that miR-146a deficiency is strongly associated with increased accumulation of leukocytes and activated kupffer cells. Interestingly, immunohistochemical staining on serial liver sections highlighted that the accumulated leukocytes and activated kupffer cells mainly contributed to increase MCP-1 production, whereas the resident liver cells in addition to leukocytes and kupffer cells contributed to the production of inflammatory cytokines such as IL-6. These observations suggests that global deficiency of miR-146a accelerates HFD-induced liver inflammation by influencing multiple cell types including hepatocytes, kupffer cells and infiltrated leukocytes. However, further studies utilizing cell-specific miR-146a deficient mice may help to understand the cellular source and mechanisms by which miR-146a regulates liver inflammation during the development of diet-induced obesity.

Although it is interesting, currently it is not clear why and how miR-146a differentially regulates inflammation in the liver and adipose tissue in the model of diet-induced obesity. In support to our current observation, a recent study in which mice fed with 45% kcal diet for either 24, 40 or 52 weeks showed that adipose tissue inflammation is established prior to hepatic inflammation, and likely to have greater contribution to insulin resistance^40^. Further, the study revealed that with 45% kcal diet, hepatic inflammation was detected only after 40 weeks whereas adipose tissue inflammation was evident as early as 24 weeks^40^. In addition, the authors observed a delayed response in the expression of inflammatory genes such as TNFα, in liver compared to adipose tissue^40^. Similarly, our study also revealed a strong response to inflammatory genes in liver compared to adipose tissue in the absence of miR-146a. The major difference in our present study is diet and duration of feeding, the 60% kcal for 16 weeks vs 45% kcal for up to 52 weeks. Although it is speculative, one possible explanation is that, during 60% kcal diet consumption for 16 weeks, adipose tissue inflammation precedes hepatic inflammation in mice, similar to that of published study^40^. Furthermore, under mild inflammatory conditions during 16 weeks of HFD, there are tissue differential responses, as in liver, miR-146a deficiency accelerated response to inflammatory genes, whereas in adipose tissue with strong inflammation, deficiency of miR-146a failed to show an accelerated response to inflammation. However, further studies are warranted to elucidate the mechanism by which miR-146a exhibits the differential response between liver and adipose tissue in diet-induced obese condition.

The major limitation of current study is the use of miR-146a germline knockout mice, as we aware of that loss or changes of a gene in one cell type, for e.g. loss of miR-146a in adipocytes, may be compensated by the changes in miR-146a in another cell types, such as macrophages or vascular stromal cells. As a result of using a germline knockout mice, we cannot certainly conclude that miR-146a has no role in adipocyte or in adipose tissue macrophage function. Therefore, generation and use of tissue-specific knockout mice such as adipocyte-specific miR-146a deficient mice will help to delineate the effect of miR-146a in adipose tissue inflammation under obese condition. Using the commercially available miR-146a floxed mice, in our future studies, we will generate adipocyte-specific miR-146a deficient mice and address its specific role in obesity.

In summary, miR-146a does not play a major role in the development of diet-induced obesity in mice. However, the functional role of miR-146a in hepatic inflammation needs to be further investigated, preferably by using different models of non-alcoholic fatty liver diseases.

## Methods

### Mice

Male miR-146a −/− (stock # 016329) mice in a C57BL/6 background were purchased from the Jackson Laboratory (Bar harbor, ME). To generate study mice, miR-146a −/− males were mated to miR-146a+/+ females (C57BL/6J; Jackson laboratory- stock # 000664) and their offspring were bred^41^ to generate miR-146a+/− males and females in a C57BL/6 background. Subsequent breeding generated relevant littermate controls of miR-146a+/+ and miR-146a −/− mice. Age-matched controls (8–10 weeks old) were used for the present study^41^. Mice were maintained in a barrier facility on a light: dark cycle of 14: 10 hours (ambient temperature of 22 °C). All study procedures were approved by the University of Kentucky Institutional Animal Care and Use Committee (Protocol # 2011–0907)^42^. This study followed recommendations from The Guide for the Care and Use of Laboratory Animals (National Institutes of Health)^42^.

### Mouse genotyping

Mouse genotypes were confirmed by PCR^42,43^. Genomic DNA was isolated from tail snips using a Maxwell tissue DNA kit (Cat# AS1030, Promega, Madison, WI). miR-146a genotyping was performed using the following primers: 5′-GCTTATGAACTT GCCTATCTGTG-3′ and 5′-CAGCAGTTCCACGCTTCA-3′. PCR of wild-type and disrupted alleles generated amplicons of 350 bp and 159 bp, respectively. A representative gel of miR-146a+/+ and −/− amplicons is shown in the Fig. 1C ^42,43^.

### Diet

Mice were fed a high fat diet (HFD, 60% kcal as fat; D12492, Research Diets Inc, New Brunswick, NJ) for 16 weeks^43^. Mice were provided water and diet *ad libitum*^43^.

### Metabolic analyses

Body weight was measured weekly. At baseline and study endpoints, fat and lean mass were measured on conscious mice using NMR spectroscopy (Echo MRI)^43^. Intraperitoneal glucose tolerance test (GTT) and insulin tolerance test (ITT) were performed after 15 weeks of HFD feeding. Mice were fasted either for 6 h (GTT) or 4 h (ITT) before intraperitoneal injections of glucose (2 g/kg body weight) or insulin (0.5 unit/kg body weight; Novolin R, Novo Nordisk Inc.). Blood glucose concentrations were measured using a glucometer at 0, 15, 30, 45, 60 and 120 minutes post injection^43^.

### Harvest of blood and tissues

At study endpoint, mice were anesthetized with a mixture of ketamine/xylazine (100/10 mg/kg, i.p) for exsanguination and tissue harvest. Blood was collected in tubes containing EDTA (0.2 mol/L), centrifuged at 2,000 rpm for 20 min (4 °C) and plasma was stored at −80 °C. Tissues were snap frozen in liquid nitrogen and stored at −80 °C^43^.

### Measurement of plasma insulin

Plasma insulin concentration was measured using commercially available ELISA kits (R&D systems) as per manufacturer instructions^43^.

### miRNA abundance

Total RNA from adipose and other tissues was extracted using the Maxwell RNA Isolation kit (Promega; catalog No: AS1340) as per manufacturer instructions. RNA (100 ng) was used for miR-cDNA synthesis using the Taqman MicroRNA reverse transcriptase (RT) kit (Taqman; catalog No: 4366596). The miR-RT synthesis kit contains RT primer specific to miR-146a (Cat No: RT000468) and snoRNA 202 (internal control, Cat No- 4427975). The RNA mixed with 1.5 ul of each of these two primers along with other reagents and subjected to RT reaction as per the kit instructions. For miR qRT-PCR, 2 ul of cDNA was used using the Taqman fast advanced master mix (Cat NO: 4444557) and the miR-146a (TM 000468) and snoRNA202 (TM001232) primers in a 15 ul reaction volume.

### mRNA abundance

Total RNA was extracted from adipose or liver tissue using the Maxwell RNA Isolation kit (Promega; catalog No: AS1340). RNA (100 ng) was reverse transcribed using the iScript cDNA synthesis kit (Cat #170–8891; Bio-Rad, Hercules, CA). Quantitative PCR was performed to quantify mRNA abundance using a SsoFas EvaGreen Supermix kit (Cat # 172–5203; Bio-Rad) on a Bio-Rad CFX96 cycler. mRNA abundances were calculated by normalization to internal control 18 S rRNA or GAPDH. Non-template and no RT reactions were used as negative controls^43^. The primers used are detailed in Supplementary Table 2.

### Liver lipid analyses

Lipids from frozen liver tissue was extracted as described previously^44^. Briefly, ~40–50 mg of liver tissue was minced and lipids were extracted with 3 ml of 2:1 chloroform: methanol (CHCl_3_:MeOH) overnight at room temperature in a glass tube with a Teflon-lined cap. Following centrifugation at 1,500 g for 10 min, the lipid extract was then transferred to new glass screw top tube and dried under nitrogen while heated at 55 °C. The dried lipid extract was dissolved in 6 ml of 2:1 CHCl_3_:MeOH with the addition of 1.2 ml of 0.05% H_2_SO_4_ for phase separation. The upper aqueous phase was removed after vigorous vortex and centrifugation as described above. An aliquot of the bottom organic phase was mixed with 2 ml 1% Trition-X100 dissolved in CHCl_3_. The samples were dried down under nitrogen and re-dissolved in 1 ml water while being heated at 60 °C for 10 min. After centrifugation at 1,500 × g for 5 min, the samples were analyzed for lipids using commercially available enzymatic kits for total cholesterol and triglycerides (Wako diagnostics). To determine the protein content, the lipid extracted liver tissue was dried and dissolved in 1 N NaOH. The protein concentration was measured using the Lowry assay^44–46^.

### *In situ* hybridization

*In situ* detection of miR-146a was performed on 5 µm paraffin embedded adipose tissue sections as described previously^47^. Briefly, slides were deparaffinized, and sections were subjected to deproteination using Proteinase K, followed by acetylation using acetic anhydride. Then the sections were prehybridized for 3 hours with the buffer (50% formamide, 5x saline sodium citrate buffer, tween-20 and 100 µg/ml yeast tRNA). For hybridization, the sections were probed with 1 µl (2.5 µM) digoxigenein labelled miRCURY LNA Detection probe complimentary to miR-146a (5′DigN/AACCCATGGAATTCAGTTCTCA/3′DigN; Exiqon Cat No: 88071–15) or scrambled miRNA (5′DigN/GTGTAACACGTCTATACG- CCCA/3′DigN; Exiqon Cat No: 99004–15) for overnight at 37 °C. After stringent washes, the sections were blocked with sheep serum (2%) and bovine serum albumin (2 mg/ml) in PBS tween for 2 hours at room temperature and probed with anti-digoxigenein- alkaline phosphatase antibody (1:500; Roche Cat No: 11093274910) for overnight at 4 °C. The *in situ* hybridization signals, bluish-purple color, were visualized using alkaline phosphatase substrates – NBT (Roche, Cat No: 11383213001)/BCIP (Roche, Cat No: 11383221001) solution. Nuclei was counterstained with nuclear fast red staining solution (Merck, Cat No: HC44369021).

### Histological analyses

Histological, immunohistochemical and immunofluorescent staining of adipose and liver tissue sections were performed on formalin-fixed sections, with appropriate negative controls, as described previously^43,48,49^. Immunohistochemical staining was performed on adipose sections to detect F4/80 positive macrophages using the rat anti-mouse F4/80 (1:200, catalog No. ab6640; Abcam, Cambridge, MA) antibodies. TUNEL staining for adipose tissue sections was performed using the *IN Situ* Cell Death Detection Kit (Roche Applied Science)^43^. Immunohistochemical staining on liver sections was performed to detect kupffer cells, CD45 positive leukocytes, MCP-1 and IL-6 using the rabbit anti-mouse IBA-1 (1:1000, catalog No. CP290B; Biocare Medical, Pacheco, CA), biotinylated rat anti-mouse CD45 (1:500, catalog No. 553077; BD Pharmingen, Franklin lakes, NJ), rabbit anti-mouse MCP-1 (1:200, catalog No. ab7202; Abcam, Cambridge) and rabbit anti-mouse IL-6 (1:500, catalog No. ab7202; Abcam, Cambridge) antibodies, respectively.

### Statistical analyses

Data are represented as mean ± SEM. Statistical analyses were performed using SigmaPlot 13.0 (SYSTAT Software Inc., San Jose, CA, USA). Student’s *t* test or Mann-Whitney Rank Sum test were performed as appropriate for two-group comparisons. The normality and equal variance distributions were tested using Shapiro-Wilk test and Brown-Forsythe test^43^. Values of *P* < 0.05 were considered to be statistically significant.

## Supplementary information

Supplementary Information

## Data Availability

All the datasets generated and/or analyzed during the current study are available upon reasonable request from the corresponding author.
