# Supplementary material for: miR-146a Deficiency Accelerates Hepatic Inflammation Without Influencing Diet-induced Obesity in Mice
Source: Sci Rep. 2019 Sep 2;9:12626. doi: 10.1038/s41598-019-49090-4 (PMC6718417; doi:10.1038/s41598-019-49090-4)

Supplementary Information

miR-146a Deficiency Accelerates Hepatic Inflammation Without Influencing Diet-induced Obesity in Mice

Aida Javidan[1], Weihua Jiang[1], Michihiro Okuyama[1], Devi Thiagarajan[1], Lihua Yang[1], Jessica J. Moorleghen[1], Latha Muniappan[1^], Venkateswaran Subramanian[1,2]*.

[1]Saha Cardiovascular Research Center, University of Kentucky, Lexington, KY, USA.
[2]Department of Physiology, University of Kentucky, Lexington, KY, USA.

^ Deceased

*** Address for Correspondence:**

Venkateswaran Subramanian
Saha Cardiovascular Research Center
Department of Physiology
BBSRB - Room 261
University of Kentucky
Lexington, KY 40536-0509
E-mail: venkat.subramanian@uky.edu

Online Supplement

Number of Figures	:	5
Number of Tables	:	1

Male

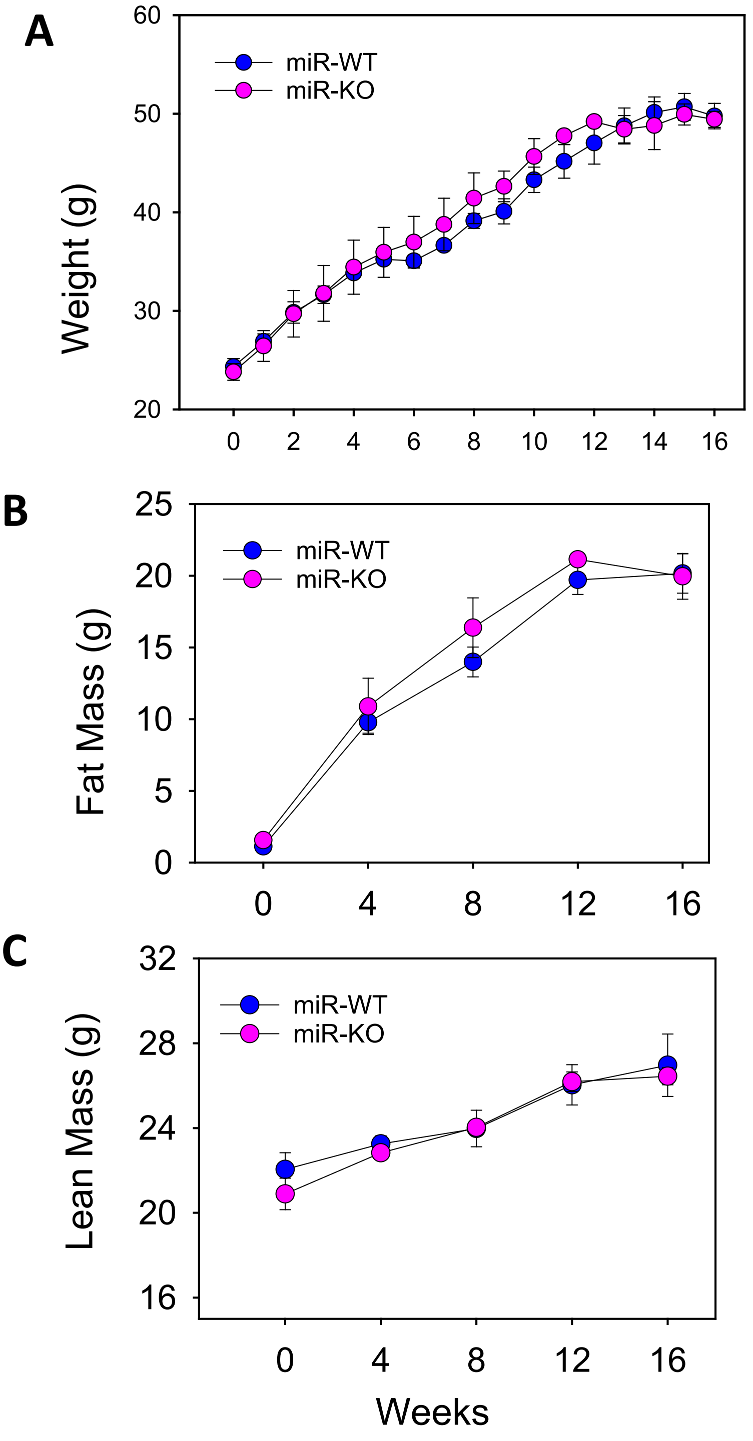

Supplementary Figure I. miR-146a deficiency had no effect on diet-induced body weight gain and fat mass in male mice.

A. Body weight of HFD fed male miR-146a WT and KO mice (n=4). Body fat mass (**B**) and lean mass (**C**) of HFD fed male miR-146a WT and KO mice (n=4). Values are represented as mean ± SEM. Statistical significance were analyzed by Student's *t* test or Mann-Whitney Rank Sum test.

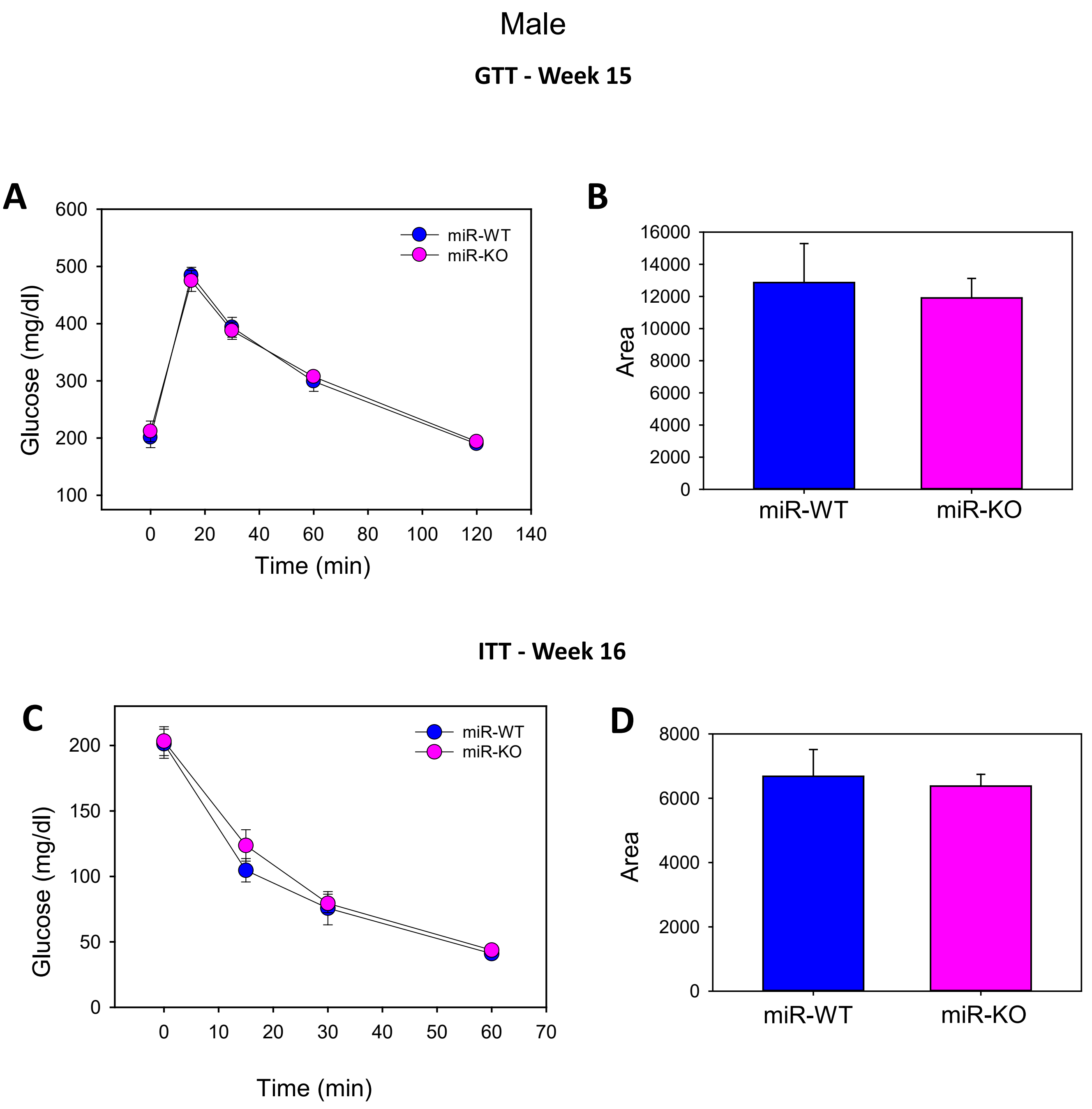

Supplementary Figure II. miR-146a does not contribute to glucose and insulin tolerance in obese male mice.

Glucose tolerance test (GTT) and the area under curve (AUC) of GTT at week 15 (**A,B**) post HFD in male miR-146a WT and KO mice. Insulin tolerance test (ITT) and the AUC of ITT at week 16 (**C,D**) post HFD in female miR-146a WT and KO mice (n=4). Values are represented as mean ± SEM. Statistical significance were analyzed by Student's *t* test or Mann-Whitney Rank Sum test.

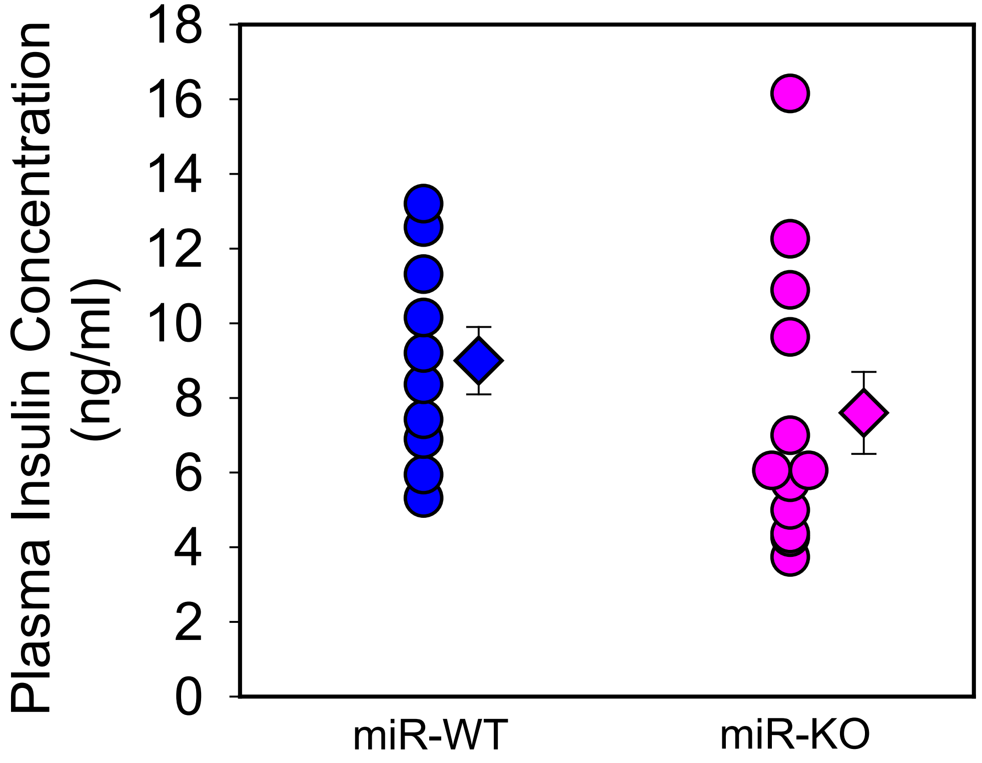

Supplementary Figure III. miR-146a deficiency had no effect on plasma insulin levels upon HFD feeding. Plasma Insulin were measured by ELISA (n=10-12). Blue (WT) and pink circles (KO) represent individual female mice, diamonds represent means, and bars are SEMs (Mann-Whitney Rank Sum test).

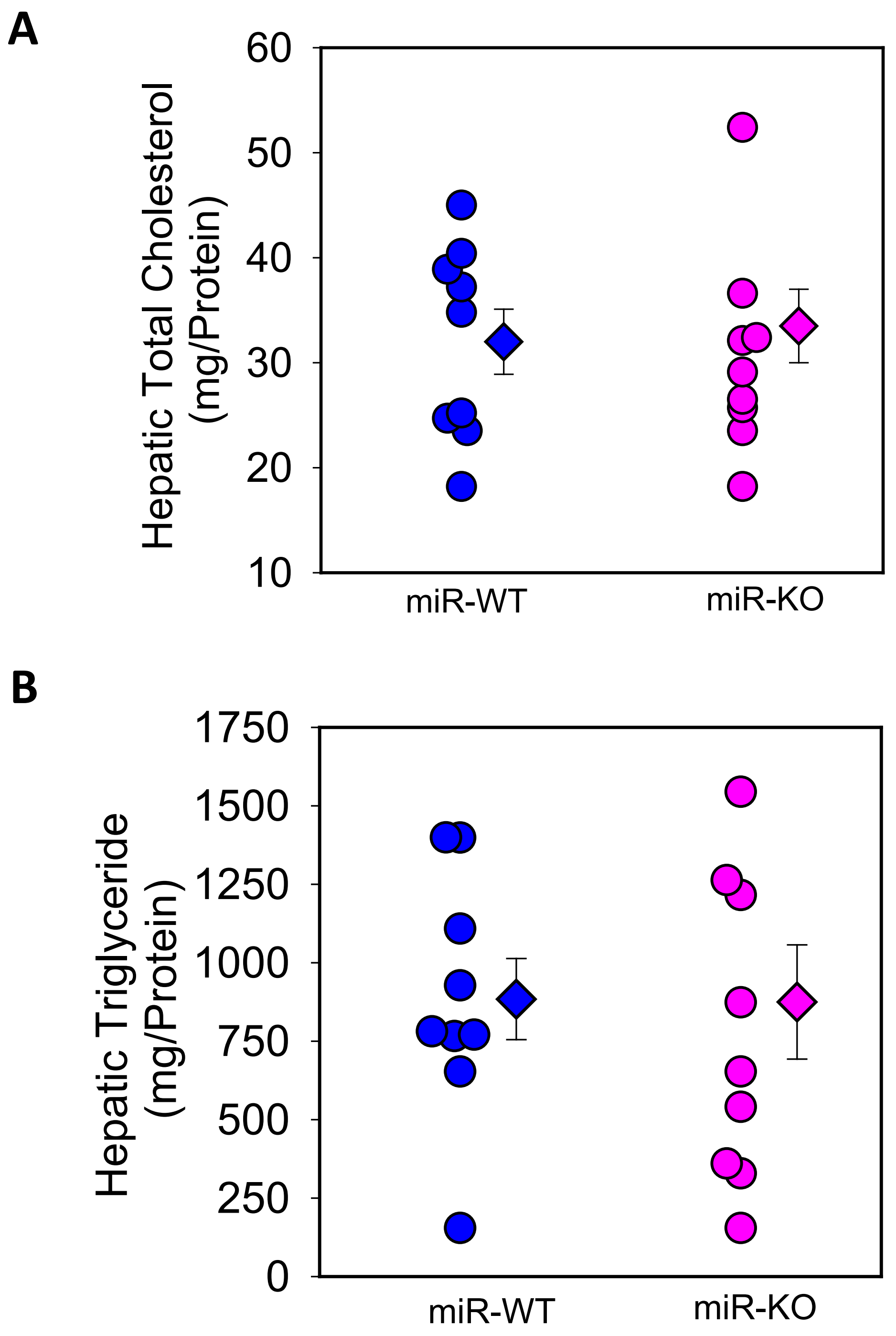

Supplementary Figure IV. miR-146a deficiency had no effect on hepatic total cholesterol and triglycerides upon HFD feeding. Concentrations of total cholesterol (**A**) and triglycerides (**B**) in the liver of HFD fed miR-146a WT and KO mice (n=9). Blue (WT) and pink circles (KO) represent individual female mice, diamonds represent means, and bars are SEMs. Statistical significance were analyzed by Student's *t* test.

A **For Biotin labelled-CD 45 Staining**

Rat IgG
Control

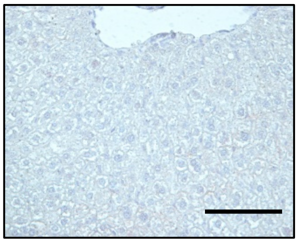

No antibodies
Control

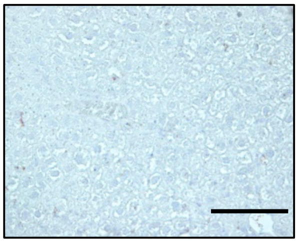

B **For IBA-1, MCP-1, IL-6 Staining**

Rabbit IgG
Control

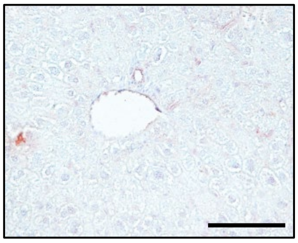

Secondary Ab –
Goat anti Rabbit
only Control

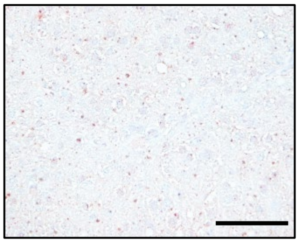

No antibodies
Control

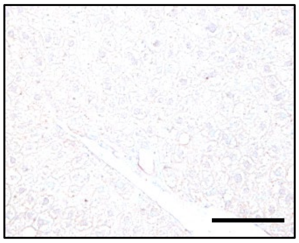

Supplementary Figure V. Representative images of negative controls for immunostaining on liver sections. Representative liver sections stained with rat IgG (**A**), rabbit IgG (**B**), only secondary antibodies (Rabbit anti-rat or Goat anti-rabbit) and no antibodies. Scale bars correspond to 50 µm (200x magnification).

Supplementary Table I. Primers used for real-time PCR

Gene	Primers	Product size (bp)
PPARγ	5’- AGCATCAGGCTTCCACTATG -3’<br>5’- ATCCGGCAGTTAAGATCACA-3’	111
CEBPα	5’-TGGACAAGAACAGCAACGAG-3’<br>5’-TCACTGGTCAACTCCAGCAC-3’	127
CEBPβ	5’-CAAGCTGAGCGACGAGTACA-3’<br>5’-AGCTGCTCCACCTTCTTCTG-3’	156
Adiponectin	5’-GGAACTTGTGCAGGTTGGAT-3’<br>5’-CGAATGGGTACATTGGGAAC-3’	293
PREF-1	5’-GGCAGTGCATCTGCAAGGAT-3’<br>5’-CAGGTCCACGCAAGTTCCAT-3’	104
Bid	5'-CTGCCTGTGCAAGCTTACTG-3'<br>5'-GTCTGGCAATGTTGTGGATG-3'	142
Bax	5'-TGTTTGCTGATGGCAACTTC-3'<br>5'-GATCAGCTCGGGCACTTTAG-3'	104
Bcl 10	5'-GCAGGGAGAAAACACAGAGC-3'<br>5'-CGGAATTGCACCTAGAGAGG-3'	156
Herpud 1	5'-CCTGGCTTCTCTGGCTACAC-3'<br>5'-GTCGGGACAAAAGTTCCTGA-3'	104
F4/80	5'-CTGTAACCGGATGGCAAACT-3'<br>5'-ATGGCCAAGGCAAGACATAC-3'	123
CD68	5'-CCAATTCAGGGTGGAAGAAA-3'<br>5'-ATGGGTACCGTCACAACCTC-3'	114
MCP-1	5'-CAGCCAGATGCAGTTAACGC -3'<br>5’-TCTGGACCCATTCCTTCTTG-3'	175
TNFα	5'-CCCACTCTGACCCCTTTACTC-3'<br>5'-TCACTGTCCCAGCATCTTGT-3'	114
IL-6	5'-GGGAAATCGTGGAAATGAGAAA-3'<br>5'-AAGTGCATCATCGTTGTTCATACA-3'	167
IL-1β	5'-TCGTGCTGTCGGACCCATAT-3'<br>5'-GTCGTTGCTTGGTTCTCCTTGT-3'	110
IL-10	5'-CCAAGCCTTATCGGAAATGA-3'<br>5'-TCTCACCCAGGGAATTCAAA-3'	190
TLR4	5'-CGCTGCCACCAGTTACAGAT-3'<br>5'-AGGAACTACCTCTATGCAGGG-3'	164

NFkB1 (P50)	5'-ACACGAGGCTACAACTCTGC-3'<br>5'-GGTACCCCCAGAGACCTCAT-3'	164
RelA (P65)	5'-CCAGACACAGATGATCGCCA-3'<br>5'-TTTCGGGTAGGCACAGCAAT-3'	143
IKKA	5'-GTCAGGACCGTGTTCTCAAGG-3'<br>5'-GCCCTCAGTAACATCAAAGAAGC-3'	118
TRAF6	5'-GATCCAGGGCTACGATGTGG-3'<br>5'-CTTGTGCCCTGCATCCCTTA-3'	157
IRAK1	5'-CATGACCCAGAGGCAAAACTC-3'<br>5'-AGCAAAGCAGCAGCCCTTTA -3'	101
Col I	5'-TGGCAACAAAGGAGACACTG-3'<br>5'-GGCTCCTCGTTTTCCTTCTT-3'	97
Col III	5'-ACCAAAAGGTGATGCTGGAC-3'<br>5'-GACCTCGTGCTCCAGTTAGC-3'	110
Col IV	5'-CCAAAGGATCAGTTGGAGGA-3'<br>5'-CTCTCCTTTGGCTCCCTTCT-3'	119
ABCA1	5'-CTGGTTTGGTGAGGAAATTCA-3'<br>5'-ACCTTCATGCCATCTCGGTA-3'	150
ABCG1	5'-GCTGGGAAGTCCACACTCAT-3’<br>5'-ATCATGGGTCCTGAAGAGT-3’	173
SREBP1	5'-GCAGACTCACTGCTGCTGAC-3’<br>5'-AGGTACTGTGGCCAAGATGG-3’	135
FABP1	5'-CAATAGGTCTGCCCGAGGAC-3’<br>5'-AGCTTGACGACTGCCTTGAC-3’	193
FASN	5'-CCAGTGTCCACCACCAAGCG-3’<br>5'-GGAGCGCAGGATAGACTCAC-3’	111
CD36	5'- TGCTGGAGCTGTTATTGGTG-3’<br>5'-TGGGTTTTGCACATCAAAGA-3’	190
G6P	5'-TGGATTCCGGTGTTTGAACG-3’<br>5'-GCAAGGTAGATCCGGGACAG-3’	84
PCK-1	5'-ATGAAAGGCCGCACCATGTA-3’<br>5'-AGGCCCAGTTGTTGACCAAA-3’	235
ACAT1	5'-GCATTCAGTGTGGTTGTGCT-3’<br>5'-AGGGCATGAGCCATATGAAC-3’	143
CD45	5'-ACCACCAGGTGAATGTCAATTT-3’<br>5'-CTTGCTTTCCCTCGGTTCTTT-3’	123
CD11C	5'-CAAAATCTCCAACCCATGCT-3'<br>5'-TCTGGGAAGCCAAATACGAC-3'	128

18S	5'-CTCTGTTCCGCCTAGTCCTG<br>5'-AATGAGCCATTCGCAGTTTC	170

Full-length blot of representative DNA genotyping gel image of Figure 1C.

Figure 1

miR-146a genotyping

c

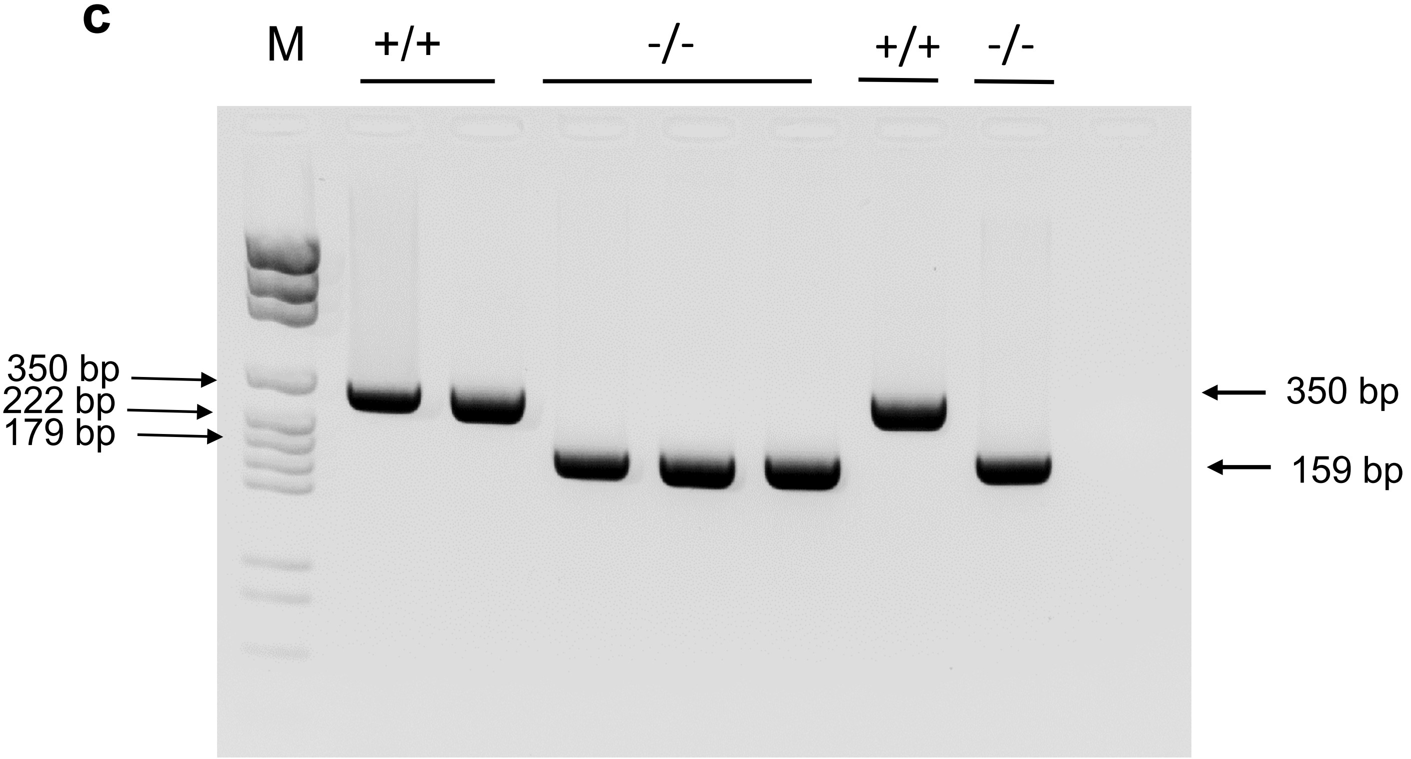

Supplement: Supplementary file 1 — Supplementary Information [file 41598_2019_49090_MOESM1_ESM.pdf]
